# Epidemiology, Clinical Features, and Outcomes of Chronic Melioidosis in the Top End of Australia's Northern Territory, 1989–2023

**DOI:** 10.1093/ofid/ofag294

**Published:** 2026-06-05

**Authors:** Harsimran Singh, Bart J Currie, Celeste Woerle, Ella M Meumann

**Affiliations:** Department of Infectious Diseases, Division of Medicine, Royal Darwin Hospital, Darwin, Northern Territory, Australia; College of Medicine and Dentistry, James Cook University, Townsville, Queensland, Australia; Department of Infectious Diseases, Division of Medicine, Royal Darwin Hospital, Darwin, Northern Territory, Australia; Global and Tropical Health Division, Menzies School of Health Research, Charles Darwin University, Darwin, Northern Territory, Australia; Global and Tropical Health Division, Menzies School of Health Research, Charles Darwin University, Darwin, Northern Territory, Australia; Department of Infectious Diseases, Division of Medicine, Royal Darwin Hospital, Darwin, Northern Territory, Australia; Global and Tropical Health Division, Menzies School of Health Research, Charles Darwin University, Darwin, Northern Territory, Australia; Microbiology Department, Territory Pathology, Darwin, Northern Territory, Australia

**Keywords:** *Burkholderia pseudomallei*, chronic infection, epidemiology, melioidosis

## Abstract

**Background:**

Melioidosis is caused by infection with *Burkholderia pseudomallei*. Most cases present acutely however a minority have chronic symptoms. We aimed to define the clinical epidemiology of chronic melioidosis.

**Method:**

Cases of melioidosis with symptoms for ≥2 months in the Australian Northern Territory from 1989 to 2023 were described and compared to cases with symptoms for <2 months.

**Results:**

Among the 126 (9.4%) chronic melioidosis cases, the most common presentations were pneumonia (38.9%) and skin and soft tissue infections (32.5%). In those with primary pneumonia, neoplasia was a differential diagnosis in 25.5% and tuberculosis in 32.4% of chest radiography reports. Patients with chronic compared to acute melioidosis were significantly more likely to have cutaneous disease (32.5% vs 10.4%, *P* < .001) and less likely to have pneumonia (38.9% vs 53.6%, *P* = .002), have positive blood cultures (14.3% vs 59.1%, *P* < .001), have more than one infective focus (13.5% vs 22.5%, *P* = .019), or die from their initial melioidosis episode (2.4% vs 11.1%, *P* = .003). Those with chronic presentations were less likely to be diagnosed in the wet season (55.6% vs 83.9%, *P* < .001), be First Nations Australians (35.7% vs 54.8%, *P* < .001), or have clinical risk factors for melioidosis including diabetes mellitus (32.5% vs 48.0%, *P* < .001), chronic kidney disease (6.4% vs 12.4%, *P* = .046), and hazardous alcohol use (27.0% vs 40.3%, *P* = .003). Serology by indirect hemagglutination assay was positive at diagnosis in 87.0% of chronic presentations compared to 48.5% of acute cases.

**Conclusions:**

Patients with chronic melioidosis have fewer risk factors for melioidosis, lower disease severity, higher serology titers, and lower mortality than patients with acute melioidosis.

## Teaser

Chronic melioidosis typically presents with cutaneous infection manifesting as nonhealing ulcers or respiratory infection, which can mimic tuberculosis or malignancy. Patients with chronic melioidosis have fewer comorbidities, higher serology titers, and lower mortality than those with acute melioidosis.

Melioidosis is the clinical infectious disease caused by *Burkholderia pseudomallei*, a Gram-negative bacterium endemic to northern Australia and other regions predominantly between the Tropics of Cancer and Capricorn [1]. Melioidosis is both increasingly being unmasked in previously suspected locations and spreading to new locations, most notably the United States[2]. The bacterium resides in soil and infects hosts through percutaneous inoculation, inhalation, or ingestion. Increased rainfall has been associated with both increased cases and a higher comparative infection severity, more often manifesting as pneumonia, hypothesized to be due to inhalation of aerosolized bacteria [3, 4]. Accordingly, a seasonal monsoonal pattern of disease has been reported in northern Australia and across Southeast Asia. The time between heavy rainfall and increased case numbers is reported to be 7–14 days, in keeping with an acute infective onset [4, 5].

Seroprevalence studies have demonstrated that while exposure to *B. pseudomallei* is relatively common in endemic areas, progression to melioidosis with clinical disease occurs in only a subset of exposed individuals; in rare cases, the bacterium can remain latent for years prior to activation [6–8]. A recent cohort study and analysis of the literature on latency of *B. pseudomallei* and subsequent activation showed that many historic cases attributed to (re)activation from latency were more likely to represent chronic melioidosis, with true reactivation accounting for under 3% of all cases [8]. Melioidosis, excluding activation from latency and relapsed disease, has previously been divided into acute, where there are symptoms for <2 months, or chronic, where symptoms have been present for at least 2 months prior to diagnosis. Acute melioidosis is most common, accounting for ∼88% of cases, while chronic infection is less common (∼9%) and has not previously been systematically characterized [9].

Infection with *B. pseudomallei* is associated with host clinical risk factors impacting immunologic function, especially innate and adaptive immunity, such as diabetes mellitus and hazardous alcohol use, as well as environmental and occupational or recreational factors such as farming, gardening, landscaping, sporting, and military activities that increase contact with the organism [3, 9]. Clinical manifestations are wide-ranging; *B. pseudomallei* can infect almost any organ system, and diagnosis cannot be made clinically due to the broad differential diagnosis associated with melioidosis presentations. Furthermore, the organism may be overlooked or misidentified in laboratories not familiar with *B. pseudomallei* [10]. Presentations with chronic melioidosis may go unrecognized due to limited clinician awareness particularly when cases arise outside endemic areas, milder disease severity, or mimicry of other infectious and noninfectious diseases.

Here, we describe the epidemiology, risk factors, clinical features, and outcomes of patients diagnosed with chronic, compared to acute, melioidosis in the Top End of the Northern Territory of Australia.

## METHODS

The Darwin Prospective Melioidosis Study (DPMS) has documented all culture-confirmed melioidosis cases in the Northern Territory Top End since 1 October 1989 [11]. This study was conducted at the Royal Darwin Hospital, the tertiary referral center for the Top End, and the hospital that manages all cases of melioidosis in the region. The Top End has a tropical savanna climate and a catchment area of 475 388 km^2^ with a current population of ∼207 000 people, 25% of whom are First Nations Australians [12, 13].

We included first episode, culture-confirmed melioidosis cases in the DPMS from 1 October 1989 to 30 August 2023. Cases were prospectively designated as chronic if the reported symptom duration was ≥2 months. The acute cohort served as a historical control for the entire study period. The minority of patients from the DPMS considered to be from activation from latency were excluded [8]. The wet season was defined as 1 November to 30 April, and the dry season was defined as 1 May to 31 October. The definitions for risk factors, clinical manifestations, and outcomes were as previously described [9] and are summarized in the Supplementary Appendix.

Serology for *B. pseudomallei* was done using the indirect hemagglutination assay (IHA) with a titer threshold of ≥1:40 used to define a positive result. The background rate of melioidosis seropositivity by IHA in the Top End has previously been reported to be 3% [14]. Admission serology was defined as within 7 days before or after hospital admission and, where multiple results were available, only the collection closest to admission was included. Electronic medical records were reviewed for collection of additional parameters related to exposures, clinical details, and radiologic findings. Core DPMS data fields were available for all cases, with the exception of some hematologic and biochemical parameters. All parameters except C-reactive protein (CRP) had <10% missing data. Multiple imputation was used to account for missing data variability.

Patient details were stored in de-identified form in a secure offline database and analyzed using International Business Machines Statistical Package for the Social Sciences Version 29.0.2.0. Univariate analyses were performed using χ^2^ tests for categorical variables, Student's or Welch's *t*-tests for categorical with numerical variables, and 1-way ANOVA with post hoc Bonferroni correction for numerical values. All numerical data were assessed for and met normality assumptions. Multivariate analysis was performed using logistic regression on specific categorical variables to calculate adjusted odds ratios. A total of 9 variables were used to avoid data overfitting and multicollinearity. Multicollinearity was assessed using the variance inflation factor (VIF); all retained variables had a VIF of <3.0, confirming no problematic collinearity. These variables were selected based on established clinical plausibility and a change-in-estimate criterion; these included First Nations status, diagnosis in wet season, clinical risk factors of diabetes mellitus, chronic kidney disease, chronic lung disease and hazardous alcohol use, presence of a bacteremia, intensive care unit (ICU) admission, and death during the initial admission. A *P* value of .05 was considered statistically significant.

The DPMS was approved by the Human Research Ethics Committee of the Northern Territory Department of Health and the Menzies School of Health Research (HREC 02/38).

## RESULTS

**Figure 1.**
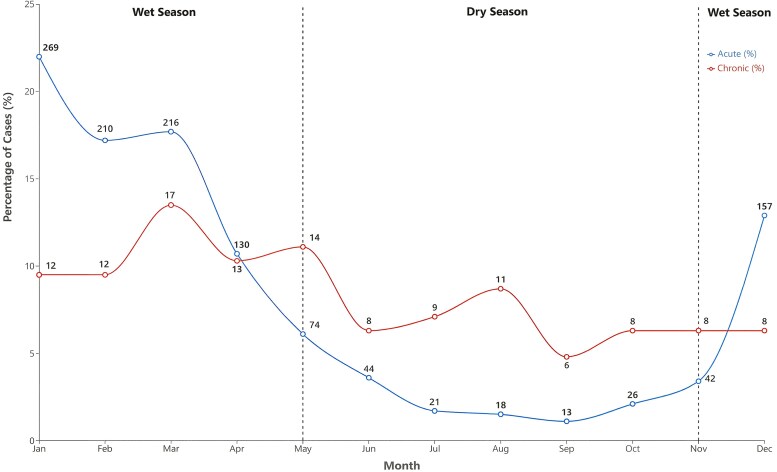
Proportion of total chronic (n = 122) and acute (n = 1220) melioidosis cases diagnosed each calendar month (absolute case numbers each month are labeled).

Of 1346 melioidosis cases between October 1989 and August 2023, 126 cases (9.36%) had a chronic presentation. The age of patients with chronic melioidosis ranged from 6 to 97 years with a mean and median of 48 years; 8 (6.35%) were children under the age of 18. There was no significant difference in mean age between patients with chronic and acute melioidosis (48.13 years [SD = 17.97] vs 49.63 years [SD = 16.89], *P* = .347). First Nations Australians were significantly less represented in the chronic than acute melioidosis cohort (35.71% vs 54.75%, *P* < .001). Seventy (55.56%) cases were diagnosed during the wet season, and 56 (44.44%) cases were diagnosed during the dry season, which was markedly different to the seasonality seen in acute melioidosis presentations (83.93% in wet season and 16.07% in dry season). The monthly distribution of diagnoses for both chronic and acute melioidosis is displayed in Figure 1.

Among patients with chronic melioidosis, the most common clinical risk factor was diabetes mellitus, followed by hazardous alcohol consumption and chronic lung disease (Table 1). Six patients were on immunosuppressive therapy, including 5 patients taking prednisolone and 1 patient taking methotrexate. In order of effect size, patients with chronic melioidosis compared to acute melioidosis were significantly less likely to have diabetes mellitus (32.54% vs 47.95%, *P* < .001), use alcohol hazardously (26.98% vs 40.33%, *P* = .003), or have chronic kidney disease (6.35% vs 12.38%, *P* = .046).

**Table 1.** Epidemiologic Features of Patients With Chronic Melioidosis (n = 126) Compared to Those With Acute Melioidosis (n = 1220) on Univariate Analysis

Characteristic	Chronic	Acute	Univariate Analysis		Multivariate Analysis	
			OR (95% CI)	*P* Value	aOR (95% CI)	*P* Value
**Seasonality**						
Wet season diagnosis	70 (55.56%)	1124 (83.93%)	.11 (.07–.16)	<.001	.31 (.20–.46)	<.001
**Demographics**					…	…
Age**^a^**	48.13 (17.97)	49.63 (16.89)	…	.347	…	…
Male sex	83 (65.87%)	753 (61.72%)	1.20 (.81–1.76)	.360	…	…
First Nations	45 (35.71%)	668 (54.75%)	.46 (.31–.67)	<.001	.74 (.48–1.14)	.171
Urban Darwin	63 (50.00%)	671 (55.00%)	.79 (.55–1.14)	.214	…	…
Rural or remote	63 (50.00%)	549 (45.00%)	1.22 (.85–1.76)	.214	…	…
**Risk factors**					…	…
Diabetes mellitus	41 (32.54%)	585 (47.95%)	.52 (.36–.77)	<.001	.92 (.59–1.43)	.711
Hazardous alcohol use	34 (26.98%)	492 (40.33%)	.55 (.36–.82)	.003	.70 (.45–1.08)	.106
Kava use	2 (1.59%)	39 (3.20%)	.49 (.12–2.04)	.314	…	…
Chronic kidney disease	8 (6.35%)	151 (12.38%)	.48 (.23–1.00)	.046	.74 (.39–1.94)	.741
Chronic lung disease	28 (22.22%)	333 (27.30%)	.76 (.49–1.18)	.221	.92 (.57–1.47)	.712
Rheumatic heart disease or congestive cardiac failure	5 (3.97%)	111 (9.10%)	.41 (.17–1.03)	.051	…	…
Malignancy	7 (5.56%)	133 (10.90%)	.48 (.22–1.05)	.061	…	…
Immunosuppressive medication	6 (4.76%)	118 (9.67%)	.47 (.20–1.08)	.070	…	…
No risk factors	31 (24.60%)	222 (18.20%)	1.47 (.95–2.26)	.080	…	…

Abbreviations: aOR, adjusted odds ratio; CI, confidence interval.

^a^Age is displayed as mean (SD) in years.

Thirty-eight (30.16%) patients with chronic melioidosis had occupational exposures to *B. pseudomallei*. Nearly all occupations were outdoor-based with soil exposure. The most common were tradespersons (21.05%), mine workers (18.42%), and farmers (10.52%). Sixty-six (52.38%) patients had a specific recreational exposure documented. Gardening was the most common exposure (34.48%), followed by sport-related soil and/or water exposures (15.15%) including football, swimming and fishing, travel to wilderness areas (13.37%), home-related nongardening soil or water exposures (10.60%), and water body and flood exposures (9.10%).

Of the 82 chronic cases with specific symptom timeframes documented, the mean duration of symptoms was 5.68 months, median 4 months, and range 3 to 36 months. Of the 126 chronic cases, 109 (86.51%) had a single clinical focus of infection, 16 (12.70%) had 2 foci and 1 (0.79%) had 3 or more foci. Compared to acute presentations, patients with chronic melioidosis were significantly less likely to have more than 1 clinical focus of infection (13.49% vs 22.45%, *P* = .019) and were significantly less likely to be bacteremic (14.29% vs 59.05%, *P* < .001) or be admitted to ICU (3.17% vs 25.46%, *P* < .001). Three (2.38%) patients died from chronic melioidosis during the initial admission, and 1 (0.79%) died from a subsequent relapse of melioidosis; initial episode mortality was significantly lower than among those with acute melioidosis (11.13%, *P* = .003). On multivariate logistic regression analysis, chronic compared to acute melioidosis was significantly associated with fewer bacteremias (adjusted odds ratio [aOR] 0.19, 95% confidence interval [CI] .11–.33, *P* < .001), fewer ICU admissions (aOR 0.28, 95% CI .10–.80, *P* = .018), and fewer wet season diagnoses (aOR 0.31, 95% CI .20–.46, *P* < .001).

The clinical presentations of patients with acute and chronic melioidosis are presented in Table 2. Two primary clinical presentations were the most common among those with chronic presentations: 49 (38.89%) patients had pneumonia and 41 (32.54%) had cutaneous disease, with a further 10 (7.94%) diagnosed with a soft tissue abscess. In addition, liver abscesses were present in 9 (7.14%) versus 49 (4.02%) and splenic abscesses in 12 (9.52%) versus 78 (6.39%) of patients with chronic and acute melioidosis, respectively.

**Table 2.** Clinical Features and Outcomes of Patients With Chronic Melioidosis (n = 126) Compared to Those With Acute Melioidosis (n = 1220) on Univariate Analysis

Characteristic	Chronic	Acute	Univariate Analysis		Multivariate Analysis	
			OR (95% CI)	*P* Value	aOR (95% CI)	*P* Value
**Primary presentations**						
Pneumonia	49 (38.89%)	653 (53.57%)	.55 (.38–.80)	.002	…	…
Cutaneous	41 (32.54%)	129 (10.41%)	4.08 (2.69–6.18)	<.001	…	…
Soft tissue abscess	10 (7.94%)	47 (3.92%)	2.15 (1.06–4.37)	.028	…	…
Genitourinary	9 (7.14%)	152 (12.41%)	.54 (.27–1.09)	.072	…	…
Bacteremia without focus	1 (0.79%)	140 (11.48%)	.06 (.01–.45)	<.001	…	…
Central nervous system	1 (0.79%)	22 (1.76%)	N/A	N/A	…	…
Septic arthritis	3 (3.28%)	32 (2.56%)	N/A	N/A	…	…
Osteomyelitis	3 (3.28%)	14 (1.20%)	N/A	N/A	…	…
Other	9 (7.14%)	31 (2.56%)	N/A	N/A	…	…
More than one clinical infective focus	17 (13.49%)	275 (22.54%)	.54 (.32–.91)	.019	…	…
**Severity**					…	…
Bacteremia	18 (14.29%)	723 (59.09%)	.11 (.07–.19)	<.001	.19 (.11–.33)	<.001
Septic shock	2 (1.59%)	271 (22.34%)	.06 (.01–.23)	<.001	…	…
ICU admission	4 (3.17%)	309 (25.46%)	.10 (.04–.26)	<.001	.28 (.10–.80)	.018
Vasoactive agents	1 (0.79%)	221 (18.10%)	.04 (.01–.26)	<.001	…	…
Ventilator	2 (1.59%)	194 (16.09%)	.09 (.02–.35)	<.001	…	…
Continuous veno-venous hemofiltration	2 (1.59%)	103 (8.57%)	.18 (.04–.72)	.006	…	…
**Outcomes**					…	…
Died due to initial melioidosis episode	3 (2.38%)	133 (11.13%)	.20 (.06–.64)	.003	.57 (.13–2.62)	.471
Subsequent relapse	7 (5.56%)	39 (3.20%)	1.78 (.78–4.07)	.165	…	…
Subsequent reinfection	3 (2.38%)	20 (1.64%)	1.46 (.43–4.99)	.541	…	…

Abbreviations: aOR, adjusted odds ratio; CI, confidence interval.

**Figure 2.**
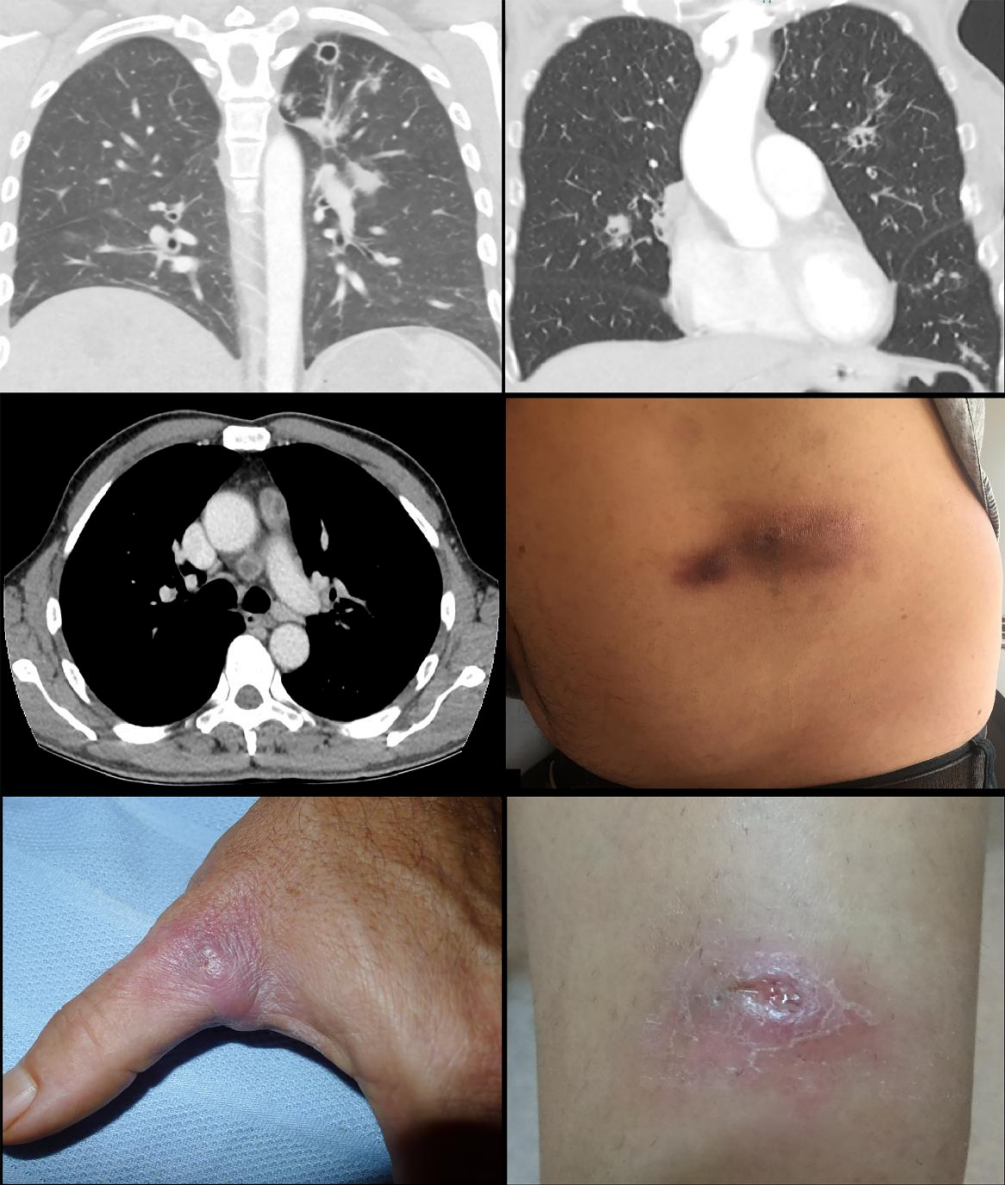
Typical clinical findings in chronic melioidosis. Top left and right: computed tomography (CT) images of cavitating pulmonary lesions. Middle left: CT image of mediastinal lymphadenopathy. Middle right, bottom left and right: cutaneous nodules and ulcer.

Of the 49 cases with a primary diagnosis of pneumonia, no cases had lobar consolidation but 21 had consolidation of a smaller region, 33 had patchy infiltrates, 16 had cavitating lesions, 19 had lymphadenopathy, and 11 had pleural effusions (Figure 2). The upper lobe(s) were involved in 32, the lower lobe(s) in 21, 14 had multilobar changes, and 10 had bilateral changes. Sites of lymphadenopathy included 11 mediastinal, 10 hilar, 6 subcarinal, 4 paratracheal, and 1 bronchial. Thirty-four (69.39%) cases had radiology reports available for review, of which 8 (23.53%) cases had neoplasia listed as a differential diagnosis and 11 (32.35%) had tuberculosis listed as a differential diagnosis. To achieve a diagnosis of melioidosis, 5 patients underwent broncho-alveolar lavage, 3 patients underwent lung biopsies, and 1 had a positron emission tomography scan and subsequent mediastinoscopy.

**Figure 3.**
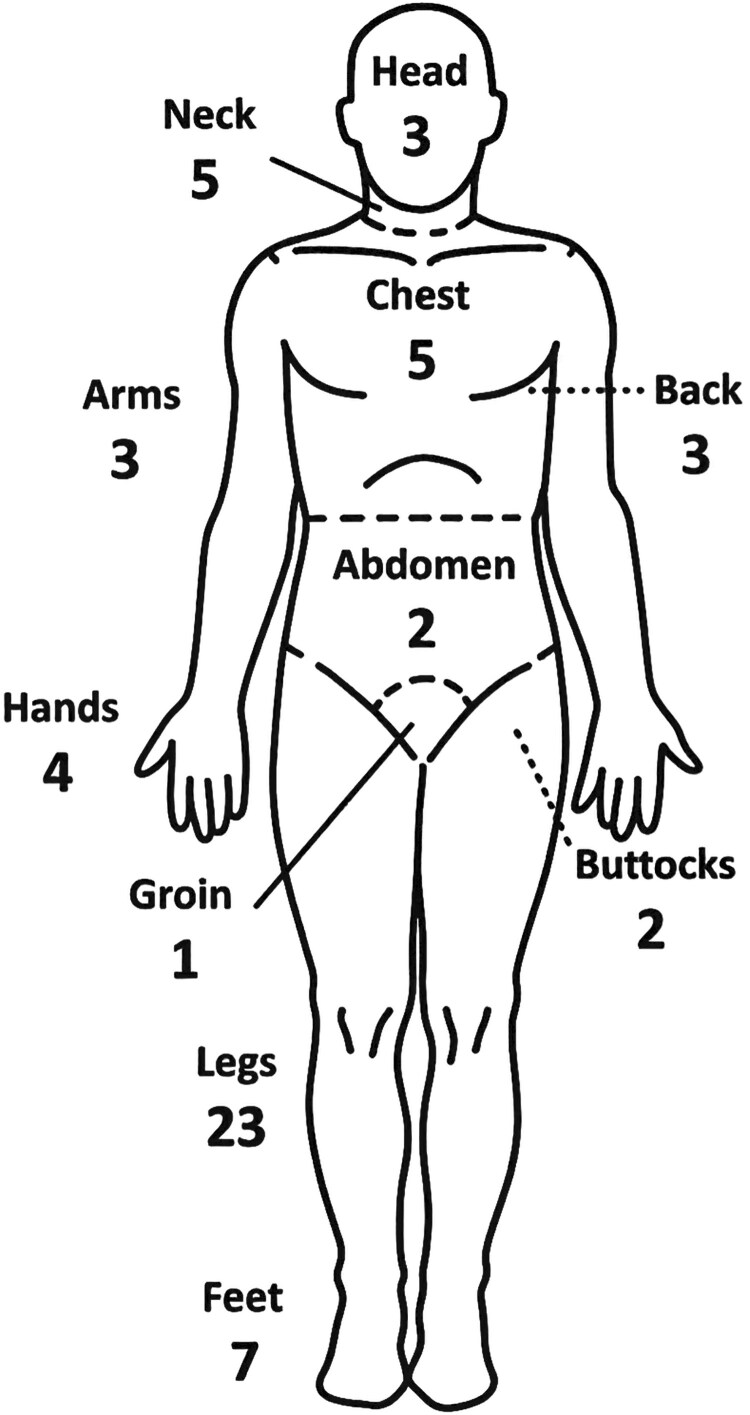
Distribution of chronic cutaneous melioidosis lesions.

Among the 41 cases with cutaneous disease, 32 involved the lower limbs, 7 the upper limbs, 10 the trunk, and 8 the head and/or neck (Figures 2 and  3). Six (14.63%) cases had more than 1 skin lesion. The most common body parts involved were the shins in 13 cases and feet in 7 cases.

**Figure 4.**
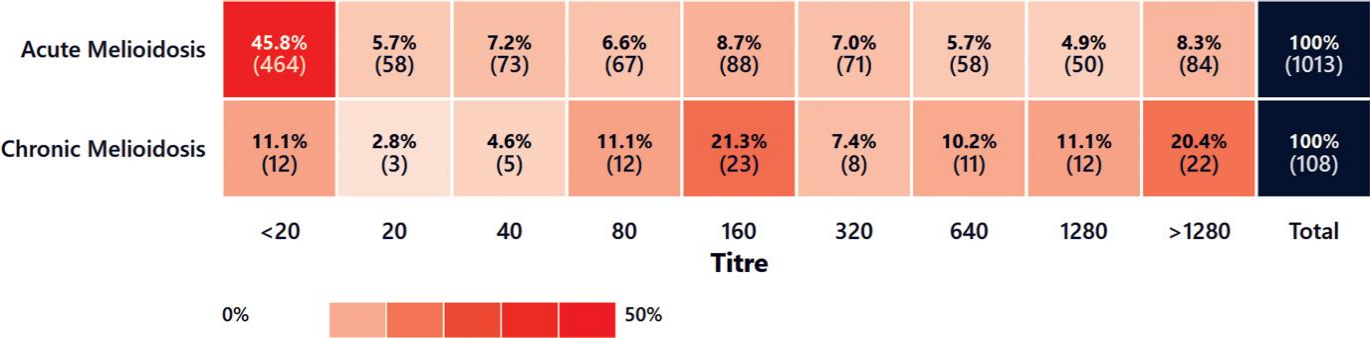
Heatmap of the proportion (%) of acute and chronic melioidosis patients with a given admission indirect hemagglutination assay (IHA) titer (absolute case numbers in brackets).

Initial IHA testing within 7 days of hospital admission was done in 108 and 1013 patients with chronic and acute melioidosis, respectively, and sensitivity was 87.04% for chronic melioidosis and 48.47% for acute melioidosis. The distribution of IHA titers is displayed in Figure 4. For chronic presentations, the median IHA titer was 1:160; 63 (58.30%) cases had a titer ≤1:320 and 45 (41.70%) cases had a titer ≥1:640. The presence of bacteremia in chronic presentations was significantly associated with a titer of ≤1:320 compared to ≥1:640 (odds ratio [OR] 1.52, 95% CI 1.16–1.99, *P* = .023). Acute cases had a median titer of 1:20; 821 (81.00%) cases had a titer ≤1:320, and 192 (19.00%) cases had titer ≥1:640. Compared to acute melioidosis, patients with chronic melioidosis were significantly more likely to present with an IHA titer of ≥1:640 (OR 3.05, 95% CI 2.02–4.62, *P* < .001).

Hematologic and biochemical parameters on admission are summarized in Table 3 along with comparisons between chronic and acute melioidosis. Within the chronic melioidosis cohort, patients with pneumonia compared to those with skin infections had a higher mean white cell count (10.59 × 10^9^/L [SD = 3.54] vs 8.69 × 10^9^/L [SD = 4.84], *P* = .035), higher mean absolute neutrophil count (8.07 × 10^9^/L [SD = 3.64] vs 5.54 × 10^9^/L [SD = 4.94], *P* = .006), lower mean absolute lymphocyte count (1.69 × 10^9^/L [SD = 0.92] vs 2.15 × 10^9^/L [SD = 0.72], *P* = .010), lower mean albumin (36.43 g/L [SD = 4.85] vs 40.76 g/L [SD = 5.60], *P* < .001), and higher mean CRP (96.36 mg/L [SD = 57.80] vs 55.40 mg/L [SD = 63.35], *P* < .001).

**Table 3.** Mean (Standard Deviation) Hematologic and Biochemical Parameters on Admission of Patients With Chronic Melioidosis (n = 126) Compared to Those With Acute Melioidosis (n = 1220) on Univariate Analysis

Laboratory Test	Chronic	Acute	*t* Value (df)	*P* Value
White cell count (10^9^/L)	9.47 (4.35)	12.97 (6.27)	−8.19 (183.45)	<.001
Neutrophil count (10^9^/L)	6.79 (4.21)	10.28 (5.95)	−8.45 (181.14)	<.001
Lymphocyte count (10^9^/L)	1.87 (1.08)	1.44 (1.23)	3.79 (1344)	<.001
Urea (mmol/L)	6.50 (7.30)	8.94 (9.14)	−3.43 (168.23)	<.001
Creatinine (µmol/L)	88.29 (65.10)	160.53 (252.26)	−7.80 (652.36)	<.001
Alanine transaminase (U/L)	31.99 (27.01)	50.66 (83.93)	−4.76 (457.09)	<.001
Bilirubin (µmol/L)	11.65 (8.20)	18.05 (25.45)	−6.20 (451.75)	<.001
Albumin (g/L)	37.68 (6.19)	35.20 (7.50)	3.60 (1344)	<.001
C-reactive protein (mg/L)	83.88 (65.08)	151.78 (101.99)	−10.46 (195.16)	<.001

## DISCUSSION

Chronic infection is an uncommon manifestation of melioidosis, accounting for 9.36% of presentations in our study. This is in keeping with other studies and is consistent with previous data published from the Northern Territory, elsewhere in tropical Australia and overseas [9, 15–17]. Patient populations were similar in age and sex, but First Nations people were relatively underrepresented in the chronic melioidosis group, potentially due to higher rates of comorbidities, such as diabetes mellitus and chronic kidney disease [18].

Compared to patients with acute presentations, there was a significantly lower prevalence of diabetes mellitus and hazardous alcohol use in patients with chronic melioidosis, and a lower but not statistically significant prevalence of chronic kidney disease, malignancies, and immunosuppressive therapy. An observed lower prevalence of risk factors in the chronic cohort compared to the acute cohort is consistent with previous findings [9, 16]. This may reflect improved capacity for containment of infection (rather than acute progression to sepsis and septic shock) in those with fewer comorbidities [8]. This is supported by the lower rates of bacteremia, septic shock, and death in those with chronic presentations and the higher number of patients presenting with localized skin and soft tissue infections. Comorbidities may also have an independent impact on disease severity and outcomes. Laboratory values in chronic compared to acute melioidosis similarly showed less inflammation and end-organ dysfunction on admission, including less lymphopenia, which has previously been associated with increased mortality in acute melioidosis [19].

Previous studies have hypothesized that higher disease severity is associated with inhalation rather than cutaneous exposure [4]. The extent to which the mode of infection (cutaneous vs inhalation) and the host immune capacity to contain infection (in the skin or lungs) contribute to acute versus chronic presentations, and disease severity, is not known. The mean duration of symptoms of nearly 6 months prior to diagnosis for this cohort with chronic melioidosis is in stark contrast to the historical melioidosis literature describing cases with many years of relapsing-remitting symptoms, partially treated by short courses of antibiotics before diagnosis [8]. It has been noted that with improved laboratory capacity to diagnose melioidosis, such protracted cases of chronic melioidosis have not been diagnosed in recent years [8].

As has been found in other countries, pneumonia was the most common manifestation of melioidosis in both acute and chronic presentations [15, 20]. Notably, no cases with chronic infection presented with lobar consolidation, which is a finding commonly reported in melioidosis [21]. Conversely, pleural involvement has previously been described as rare but was relatively common in our chronic melioidosis cohort, occurring in 22.45% of patients who presented with pneumonia [21]. Cavitating lesions and lymphadenopathy were also common findings and tuberculosis was a differential diagnosis in nearly one-third of available radiology reports, which is especially relevant to our setting with the highest incidence of tuberculosis in Australia [22]. Neoplasia was also a common differential diagnosis and mentioned in 23.53% of radiology reports. Importantly, over 18% of patients required an invasive investigation, including bronchoscopies, lung biopsies and a mediastinoscopy, to establish the diagnosis of chronic melioidosis, which reflects the difficulty of diagnostics in cases without bacteremia, who may or may not be able to produce sputum. Melioidosis has frequently been termed “the great masquerader” in the literature, and this applies particularly to chronic cases [23, 24].

Indirect hemagglutination assay titers were significantly higher on admission in patients with chronic compared to acute infection, reflecting evolution of the immune response over a longer duration of infection. Due to the low sensitivity in early infection, IHA is generally considered a poor diagnostic test for melioidosis, as evidenced by the median titer of 1:20 in the acute melioidosis cohort. However, our findings suggest that in chronic infection, it can be an important diagnostic clue. Given background seropositivity is lower in the Top End compared to some other melioidosis-endemic settings (3% vs 20%–30% in Eastern India and 60%–70% in northern Thailand), the positive predictive value of a raised IHA titer is higher in our setting [7, 14, 25, 26]. Positive serology prompts clinical evaluation and investigation for melioidosis and can prompt use of Ashdown's selective media by the laboratory for isolation of *B. pseudomallei* from specimens from nonsterile sites, increasing the yield from these and potentially precluding the need for invasive investigations [10].

In tropical areas, most melioidosis is diagnosed during the monsoonal wet season. Heavy rainfall and flooding are associated with increased melioidosis incidence, as the bacteria rise with the water table and multiply [27]. However, we found that chronic compared to acute melioidosis diagnoses were more evenly distributed throughout the year. A median symptom duration of 4 months prior to diagnosis suggests that most patients with chronic melioidosis diagnosed in the dry season were likely infected during the preceding wet season.

Strengths of this study include prospective, complete, and continuous data collection, the large cohort size, and relevance to clinicians in recognizing the features of this condition. There were several study limitations. The original classifications of chronic cases are at risk of recall bias. Cases occurring prior to 2000 were limited in access to certain relevant data points including radiologic findings, requiring case omission for those specific analyses. Fixed dates for the wet season were used for the purposes of the study; however, there is year-to-year variation (including locally across the Top End), particularly in shoulder months. Coinfections and any possible impacts on clinical features and outcomes were not considered in the analysis.

In conclusion, chronic presentations of melioidosis, defined by symptoms lasting 2 months or longer prior to diagnosis, are uncommon. Compared to acute melioidosis, these cases occur in less comorbid patients with a lower frequency of traditional risk factors for melioidosis and are not uncommonly diagnosed during the dry season. Patients are more likely to have an indolent course with a higher frequency of skin and soft tissue infections. Serology is more likely to be positive and significantly more likely to be a high titer ≥1:640. Patients are more likely to have localized rather than disseminated disease, and disease severity and mortality are lower. These findings may help clinicians to recognize chronic presentations of melioidosis, differentiate it from tuberculosis and malignancy, assist with risk stratification, and reduce unnecessary invasive investigations.

## Supplementary Material

ofag294_Supplementary_Data

## Supplementary Data

Supplementary materials are available at *Open Forum Infectious Diseases* online. Consisting of data provided by the authors to benefit the reader, the posted materials are not copyedited and are the sole responsibility of the authors, so questions or comments should be addressed to the corresponding author.
