# Supplementary material for: Epidemiology, Clinical Features, and Outcomes of Chronic Melioidosis in the Top End of Australia's Northern Territory, 1989–2023
Source: Open Forum Infect Dis. 2026 Jun 5;13(6):ofag294. doi: 10.1093/ofid/ofag294 (PMC13241206; doi:10.1093/ofid/ofag294)
Supplement: ofag294_Supplementary_Data [file ofag294_supplementary_data.docx]

**SUPPLEMENTARY APPENDIX**

**Definitions for demographic, clinical risk factors and clinical illness parameters**

Patient location was based on residence or a known likely location when infection occurred. Clinical risk factors and clinical illness parameters used constant definitions over the 30 years. Variables recorded were age, sex, ethnicity (First Nations Australian or other), and the previously identified clinical risk factors of diabetes, hazardous alcohol use, chronic renal disease and chronic lung disease. Hazardous alcohol use was defined as greater than an average daily consumption of six standard drinks (60 g alcohol total) for males and four (40 g alcohol total) for females. Chronic renal disease was defined as a creatinine of >150umol/L (N. R. <90umol/L) before the admission with melioidosis, or after completion of therapy if not previously documented. Chronic lung disease was defined as a documented diagnosis of chronic obstructive airways disease or bronchiectasis. Recent or current malignancy, immunosuppressive illness or immunosuppressive therapy, confirmed rheumatic heart disease or congestive cardiac failure and a history of recent kava ingestion were also documented. “No clinical risk factors” referred to any patient with none of the above presumptive clinical risk factors.

Each patient was assigned to a single primary clinical diagnosis on presentation, representing the dominant organ involvement on clinical assessment by the Infectious Diseases team: pneumonia, skin infection without systemic symptoms, genitourinary infection, bacteremia with no evident focus, soft tissue abscess(es) either subcutaneous and/or lymph node, septic arthritis, osteomyelitis, neurological melioidosis and other. Presence or absence of bacteremia was recorded. Septic shock was defined as the presence of hypotension not responsive to fluid replacement, together with hypoperfusion abnormalities manifest as end organ dysfunction (American College of Chest Physicians/Society of Critical Care Medicine Consensus Conference: definitions for sepsis and organ failure and guidelines for the use of innovative therapies in sepsis. Crit Care Med 1992;20: 864–874). This historical definition of septic shock was used throughout the study period and, for this analysis, contemporary pneumonia and sepsis severity scores such as the pneumonia severity index (PSI) and the APACHE score were not used, having not been in use when this prospective study commenced.

Relapsed infections were defined as recurrence after completion of antibiotic eradication therapy with the same *B. pseudomallei* isolate whilst reinfections occurred after completion of eradication therapy with a different *B. pseudomallei* isolate, as determined by the epidemiology and comparative genomics, using multilocus-sequence typing and/or whole-genome sequencing.
